# Association Between Endometriosis and Autoimmune Thyroid Disease Using a Multicenter Observational Medical Outcomes Partnership (OMOP) Common Data Model

**DOI:** 10.3390/jcm15176919

**Published:** 2026-09-07

**Authors:** Eun Hee Yu, Hyun Joo Lee, Young Mi Han, Jong Kil Joo

**Affiliations:** 1 Department of Obstetrics and Gynecology and Biomedical Research Institute, Pusan National University Hospital, Busan 49241, Republic of Korea; hoyyeh@naver.com (E.H.Y.); atouchof@pusan.ac.kr (H.J.L.); 2 Department of Obstetrics and Gynecology, School of Medicine, Pusan National University, Busan 49241, Republic of Korea; 3 R&D Team, Visual Terminology Co., Ltd., Busan 49241, Republic of Korea; maryhan01@gmail.com

**Keywords:** endometriosis, autoimmune thyroid disease, common data model, propensity score matching, real-world evidence

## Abstract

**Background**: Endometriosis affects approximately 6–10% of women of reproductive age—an estimate that varies substantially with the diagnostic standard applied—and is increasingly recognized as a systemic inflammatory disease. Growing evidence implicates shared immunological pathways between endometriosis and autoimmune thyroid disease, yet large-scale real-world evidence from standardized multi-site data remains limited. We evaluated the association between endometriosis and newly recorded autoimmune thyroid disease using federated Observational Medical Outcomes Partnership (OMOP) Common Data Model (CDM) data from 12 Korean hospitals. **Methods**: We conducted a propensity score (PS)-matched cohort study using OMOP-CDM version 5.3 data from 12 Korean tertiary academic medical centers. Women aged 18–60 years with a first recorded endometriosis diagnosis were matched 1:1 to controls without endometriosis on age, calendar year, hypertension, and selected Charlson comorbidity index components. Site-specific Cox proportional hazards models, fitted to patient-level records locally at each institution, estimated hazard ratios (HRs) for newly recorded autoimmune thyroid disease, defined as Hashimoto’s thyroiditis or Graves’ disease. Pooled estimates were derived using DerSimonian–Laird random-effects meta-analysis; leave-one-out meta-analysis, meta-regression on site-specific follow-up ratio, and an E-value were used to assess robustness. **Results**: After 1:1 PS matching, 71,619 women with endometriosis and 71,619 matched controls were included. Mean follow-up was 2693 days in the endometriosis group and 1677 days in the control group. A directionally positive but statistically inconclusive association was observed between endometriosis and autoimmune thyroid disease (pooled HR 1.22, 95% CI 0.97–1.53; *p* = 0.090; I^2^ = 38.2%). Seven of twelve sites reported HRs above 1.0, including two sites reaching individual statistical significance: AUMC (HR 1.38, *p* = 0.030) and KHUH (HR 2.69, *p* < 0.001). The pooled HR remained above 1.0 in all 12 leave-one-out iterations (range 1.17–1.31); omission of KHUH reduced I^2^ to 6.6%. Site-specific follow-up imbalance was not associated with the site-specific log HR (meta-regression *p* = 0.887). The E-value for the point estimate was 1.74. **Conclusions**: In this multi-site OMOP-CDM analysis, endometriosis showed a directionally positive but statistically inconclusive association with newly recorded autoimmune thyroid disease. The confidence interval is compatible with both no association and a clinically meaningful increase in risk, and the findings are hypothesis-generating rather than confirmatory. They do not support any change in the clinical evaluation of women with endometriosis. Further validation is warranted using standardized outcome definitions, thyroid autoantibody measurements, and thyroid function tests.

## 1. Introduction

Endometriosis, defined by the presence of endometrial-like tissue outside the uterine cavity, affects an estimated 6–10% of women of reproductive age worldwide and is associated with chronic pelvic pain, dysmenorrhea, and infertility [1,2]. In Korea, national health insurance data estimate the prevalence of endometriosis at approximately 1.7–2.0% of reproductive-age women, with an increasing trend in incidence over recent decades [3]. Beyond its gynecological manifestations, endometriosis is increasingly recognized as a systemic inflammatory disease characterized by elevated peritoneal cytokines, aberrant natural killer cell activity, and impaired immune surveillance [4,5]. Contemporary reviews emphasize that prevalence estimates vary substantially with the diagnostic standard applied—surgical, imaging-based, or code-based—and that code-based ascertainment, as used in the present study, captures clinically recorded rather than true underlying prevalence [6,7].

Among the systemic consequences of endometriosis-associated immune dysregulation, its relationship with autoimmune thyroid disease—specifically Hashimoto’s thyroiditis and Graves’ disease—has attracted growing interest. A systematic review and meta-analysis reported a non-significant increase in autoimmune thyroid disease risk in women with endometriosis, attributing imprecision to high between-study heterogeneity [8]. Epidemiological studies have reported higher prevalences of thyroid autoimmunity among women with endometriosis [9], and prior analysis using Korean national health insurance data demonstrated a significant association with Graves’ disease (OR 2.52; 95% CI 1.30–4.88) [10]. However, these findings require validation using standardized, multi-source data.

The OMOP Common Data Model (CDM) enables federated, distributed analysis across heterogeneous electronic health record systems without sharing patient-level data, providing a rigorous framework for multi-site real-world evidence generation [11,12]. Compared with single national claims databases, OMOP-CDM analyses offer the additional advantage of site-level heterogeneity assessment through meta-analytic pooling, allowing evaluation of result consistency across diverse clinical settings.

We therefore conducted a multi-site, propensity score-matched cohort study using OMOP-CDM data from 12 Korean tertiary hospitals to evaluate the association between endometriosis and newly recorded autoimmune thyroid disease. We hypothesized that endometriosis would be associated with a higher likelihood of newly recorded autoimmune thyroid disease, consistent with shared immunological dysregulation.

## 2. Materials and Methods

### 2.1. Study Design and Data Sources

**Figure 1:**
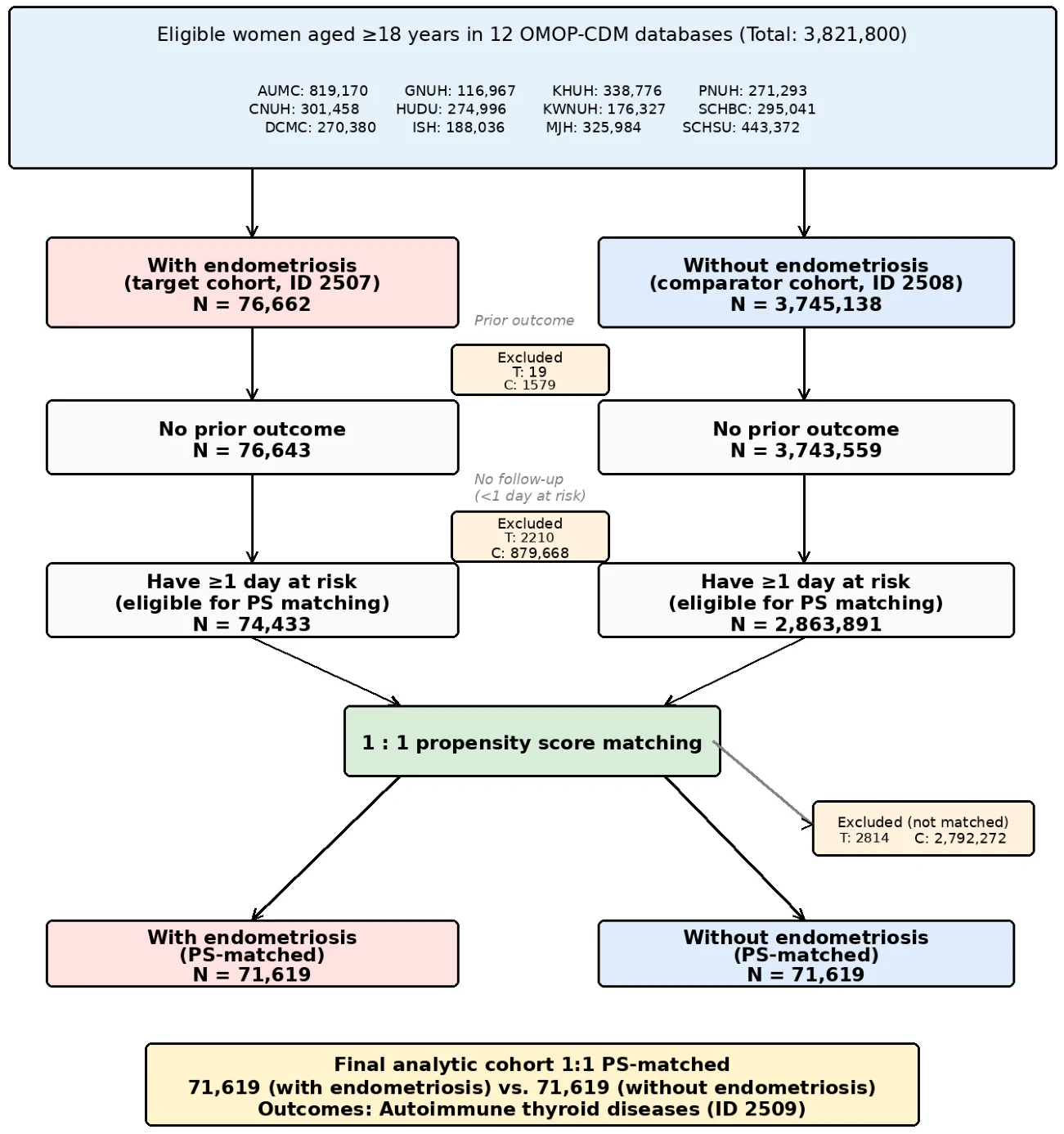
Study flow diagram. Schematic overview of cohort identification, propensity score matching, and final analytic sample across 12 OMOP-CDM participating sites. The **left** branch shows the target cohort (women with endometriosis) and the **right** branch shows the comparator cohort (women without endometriosis). Numbers shown reflect the total across all sites. OMOP-CDM = Observational Medical Outcomes Partnership Common Data Model; PS = propensity score. T = target cohort (women with endometriosis); C = comparator cohort (women without endometriosis). Numbers in the exclusion boxes indicate participants removed from each cohort at that step.

**Figure 2:**
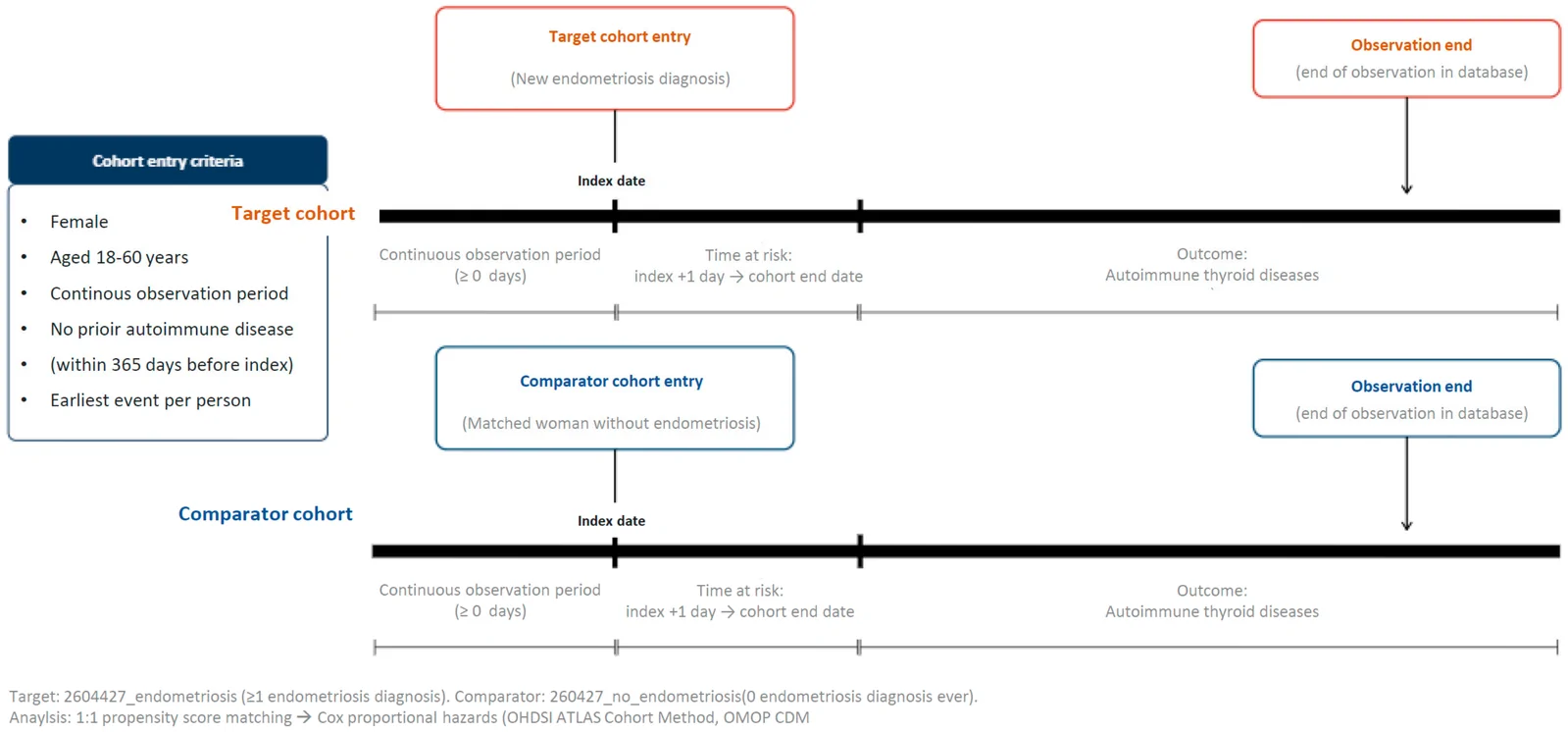
Study design—cohort construction. Schematic representation of the time-at-risk design for the target cohort (women with endometriosis, **upper** panel) and the comparator cohort (matched women without endometriosis, **lower** panel). The index date is defined as the date of first recorded endometriosis diagnosis for the target cohort and a randomly selected outpatient visit date for the comparator cohort. Time-at-risk begins at index date +1 day and extends to the earliest of outcome event or end of observation. The primary outcome is newly recorded autoimmune thyroid disease. Cohort entry criteria are shown in the **left** panel. Target cohort: 260427_endometriosis (≥1 endometriosis diagnosis). Comparator cohort: 260427_no_endometriosis (0 endometriosis diagnoses ever). Analysis: 1:1 propensity score matching -> Cox proportional hazards (OHDSI ATLAS Cohort Method, OMOP CDM).

We conducted a propensity score-matched retrospective cohort study using OMOP-CDM version 5.3 data from 12 Korean tertiary academic medical centers. The participating hospitals, site abbreviations, and site-specific OMOP-CDM data availability periods are provided in Table S1. Because the available data period differed across hospitals, eligible patients were identified within each site-specific data availability period using a common analytic protocol. All analyses were executed locally at each site, and only aggregate summary statistics were shared for pooling. The study flow is summarized in Figure 1, and the time-at-risk design is illustrated in Figure 2. Analyses were executed in a fully distributed manner: a common analytic script was run locally against the patient-level records held within each institution’s CDM instance, and only aggregate site-level summary statistics—not patient-level or event-level data—were returned to the coordinating center for meta-analytic pooling. No patient-level data were transferred at any stage. The study is reported in accordance with the STROBE and RECORD statements [13].

### 2.2. Cohort Definition

The target cohort comprised women aged 18–60 years with a first recorded diagnosis code for endometriosis within each site-specific OMOP-CDM data availability period. Endometriosis was identified using an OMOP concept set derived from KCD N80.x codes. Because the OMOP-CDM data used in this study were based on structured diagnostic codes, the exposure definition reflects clinically recorded endometriosis and does not necessarily indicate surgically or histologically confirmed disease. Patients with a recorded history of autoimmune thyroid disease within 365 days before the index date were excluded. The comparator cohort comprised women in the same age range with no recorded diagnosis code for endometriosis at any point during the available observation period, with the same baseline exclusion for autoimmune thyroid disease applied. The index date was defined as the date of the first recorded endometriosis diagnosis code for the target cohort and as a randomly selected eligible outpatient visit date for the comparator cohort. Endometriosis was ascertained solely from structured KCD7 N80.x diagnosis codes. Neither surgical nor histopathological confirmation was required or available, and disease stage, lesion subtype, and anatomical distribution could not be determined; although the N80 subcategories nominally distinguish anatomical sites (Table S2), unspecified coding predominates in Korean practice, making subtype classification unreliable. The exposure therefore denotes clinically recorded endometriosis and is expected to include some proportion of clinically suspected but unconfirmed disease. Gravidity, parity, menstrual pattern, marital status, and body mass index are not populated with usable cross-site completeness in the participating CDM instances and could not be characterized.

### 2.3. Propensity Score Matching

Within each participating site, propensity scores were estimated using logistic regression with covariates measured during the 365 days before the index date. These covariates included age as a continuous variable, index calendar year, hypertension, and selected Charlson comorbidity index components, including cerebrovascular disease, chronic pulmonary disease, peptic ulcer disease, diabetes mellitus, diabetes with chronic complications, renal disease, any malignancy, mild liver disease, and moderate-to-severe liver disease. Participants were matched 1:1 on the logit of the propensity score using a caliper of 0.2 standard deviations [14,15]. Covariate balance was assessed using standardized mean differences (SMDs), with an SMD < 0.10 considered indicative of adequate balance. The covariate set was deliberately restricted to variables that are reliably populated across all 12 participating institutions and mapped to standard OMOP concepts with comparable completeness at every site. In a federated design, a covariate that is well captured at some sites and sparsely captured at others introduces site-dependent differential adjustment, which would confound the between-site comparison that is the methodological purpose of this study. Parity and gravidity were unavailable at all sites; body mass index and smoking status reside in the Measurement and Observation domains and are populated principally through health-screening encounters, with completeness varying by more than an order of magnitude across sites; and socioeconomic indicators are not represented in hospital-derived CDM instances. Hormonal medication use was not included because progestins, combined oral contraceptives, and GnRH agonists are first-line treatments for endometriosis itself, so that adjustment would remove part of the exposure. Prevalent autoimmune comorbidity was likewise excluded, as it may lie on the causal pathway between systemic inflammatory dysregulation and thyroid autoimmunity. Residual confounding from these sources was instead bounded quantitatively using the E-value (Section 2.5).

### 2.4. Outcome Definition

The primary outcome was newly recorded autoimmune thyroid disease, defined as the first occurrence of autoimmune thyroiditis (OMOP concept ID 42485936, KCD7) or Graves’ disease (thyrotoxicosis with diffuse goiter; OMOP concept ID 42485924, KCD7) after the index date +1 day, with all descendant codes included. Full concept set definitions are provided in Table S2. The composite outcome was pre-specified on the basis of shared autoimmune etiology and of event counts adequate for site-level estimation; we acknowledge that it may obscure subtype-specific effects, and Hashimoto’s thyroiditis and Graves’ disease are enumerated separately in Table S2 so that its composition is transparent. Thyroid medications, including levothyroxine and antithyroid drugs, were not used to define or augment the outcome. As for the exposure, the outcome was ascertained from diagnosis codes alone: no thyroid autoantibody titer (anti-TPO, anti-thyroglobulin, or TSH receptor antibody) or thyroid function measurement (TSH, free T4) was required or available for outcome adjudication.

### 2.5. Site-Level Analysis and Meta-Analytic Pooling

Within each site, time-to-event analysis was performed using Cox proportional hazards regression. Time-at-risk extended from index date +1 day to the earliest of outcome event, end of observation period, or death, where available in the OMOP-CDM data. Incidence rates (IRs) were calculated per 1000 patient-years. Site-specific log hazard ratios were pooled using DerSimonian–Laird random-effects meta-analysis [16]. Between-site heterogeneity was assessed using the I^2^ statistic and Cochran’s Q test [17]. All analyses were conducted using the OHDSI Cohort Method package and Python (v3.10.11) within the OMOP-CDM analytical framework [18]. Three post hoc robustness analyses, undertaken in response to peer review, were performed on the site-specific estimates already obtained from the primary analysis. First, a leave-one-out random-effects meta-analysis was conducted by sequentially omitting each of the 12 sites and recomputing the pooled hazard ratio and I^2^. Second, to assess surveillance bias arising from differential follow-up, a random-effects meta-regression of site-specific log hazard ratios on the site-specific ratio of mean follow-up time (endometriosis: comparator) was fitted, with Spearman correlation as a non-parametric check. Third, an E-value was calculated for the pooled estimate and for the confidence interval limit closest to the null, quantifying the minimum strength of association that an unmeasured confounder would require, with both exposure and outcome, to explain away the observed result [19].

## 3. Results

### 3.1. Study Population and Baseline Characteristics

Across 12 sites, 74,433 women with endometriosis were identified, of whom 71,619 (96.2%) were successfully matched 1:1 to controls (Table 1). After PS matching, the age distribution was comparable between groups across all sites, with the majority of participants in the 35–44 age range. Before matching, SMDs for several age groups exceeded 0.10; after matching, all age-group SMDs were reduced below 0.10, confirming adequate balance. Baseline comorbidities included in the propensity score model were uniformly low in both cohorts after matching. Hypertension was present in 392 (0.55%) endometriosis subjects versus 280 (0.39%) control subjects; diabetes mellitus in 249 (0.35%) versus 190 (0.27%); renal disease in 40 (0.06%) versus 24 (0.03%). All post-matching SMDs for baseline comorbidities were below 0.10, confirming well-balanced cohorts.

### 3.2. Follow-Up and Incidence Rates

Mean follow-up was substantially longer in the endometriosis cohort (pooled: 2693 days; site range 1342–4103 days) compared with controls (pooled: 1677 days; site range 875–2622 days), a pattern consistent across all 12 sites. Total outcome events were 800 in the endometriosis group and 435 in controls, with pooled incidence rates of 1.51 and 1.32 per 1000 patient-years, respectively (Table 2).

### 3.3. Risk of Autoimmune Thyroid Disease

**Figure 3:**
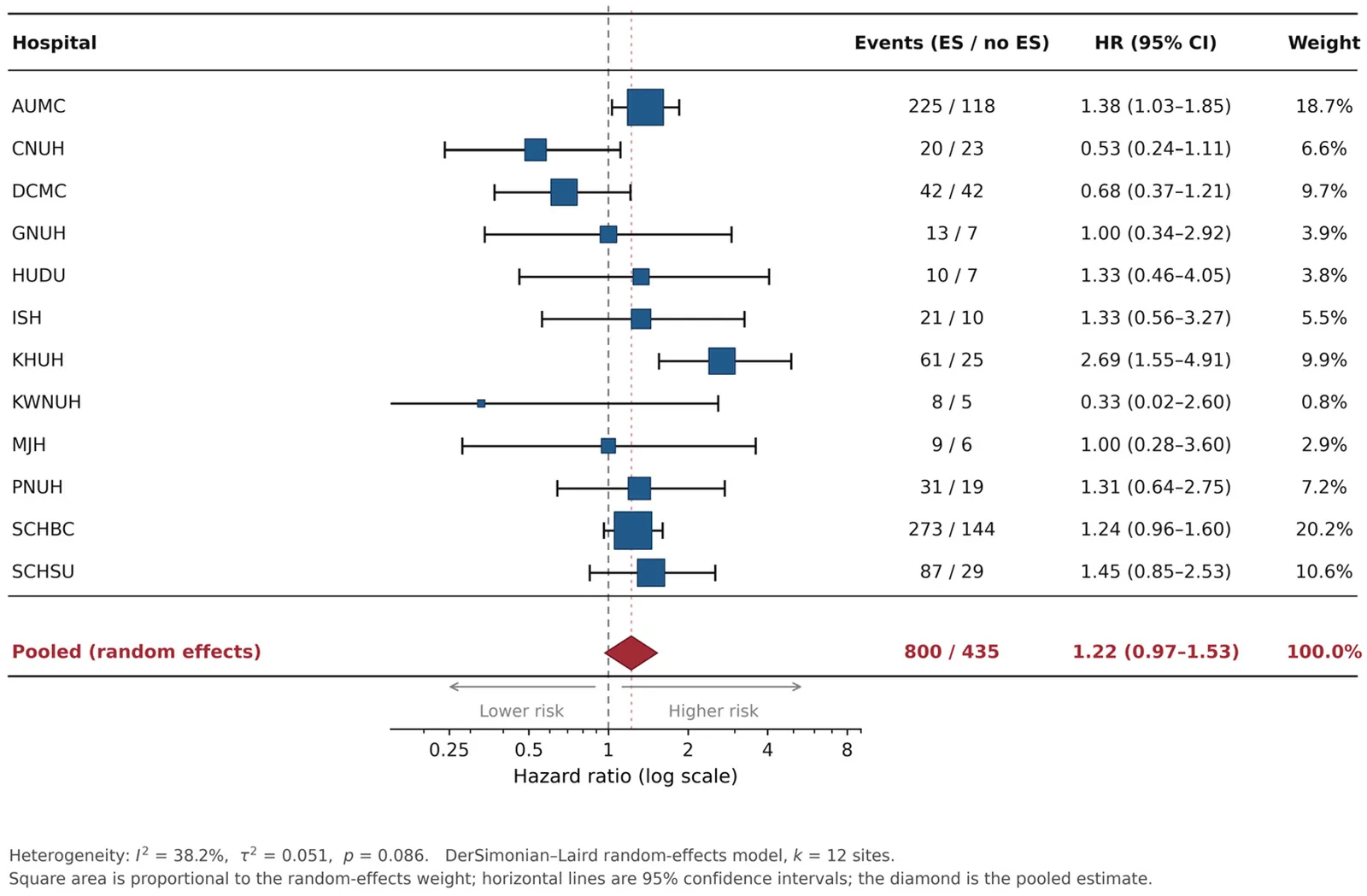
Forest plot of site-specific and pooled hazard ratios for autoimmune thyroid disease. Hazard ratios from Cox proportional hazards regression in 1:1 propensity score-matched cohorts are shown for each of the 12 participating sites (endometriosis vs. no endometriosis). Squares represent site-specific point estimates with area proportional to the random-effects inverse-variance weight; horizontal lines represent 95% confidence intervals. The diamond represents the pooled random-effects hazard ratio derived by DerSimonian–Laird meta-analysis. The dashed vertical line indicates HR = 1.0 (null effect). Between-site heterogeneity statistics are shown below the plot. The number of outcome events observed in each cohort (ES/no ES) is tabulated alongside each estimate, and the hazard ratio axis is logarithmic. HR = hazard ratio; ES = endometriosis.

A directionally positive but statistically inconclusive association with autoimmune thyroid disease was observed in the endometriosis cohort across sites (pooled HR 1.22, 95% CI 0.97–1.53; *p* = 0.090; I^2^ = 38.2%; Figure 3). Although the pooled estimate did not reach conventional statistical significance, 7 of 12 sites reported HRs above 1.0. Two sites individually reached statistical significance: AUMC (HR 1.38, 95% CI 1.03–1.85; *p* = 0.030) and KHUH (HR 2.69, 95% CI 1.55–4.91; *p* < 0.001). Four sites contributed fewer than 20 outcome events in total across both cohorts (KWNUH 13, MJH 15, HUDU 17, and GNUH 20), and their site-specific estimates are correspondingly imprecise.

**Figure 4:**
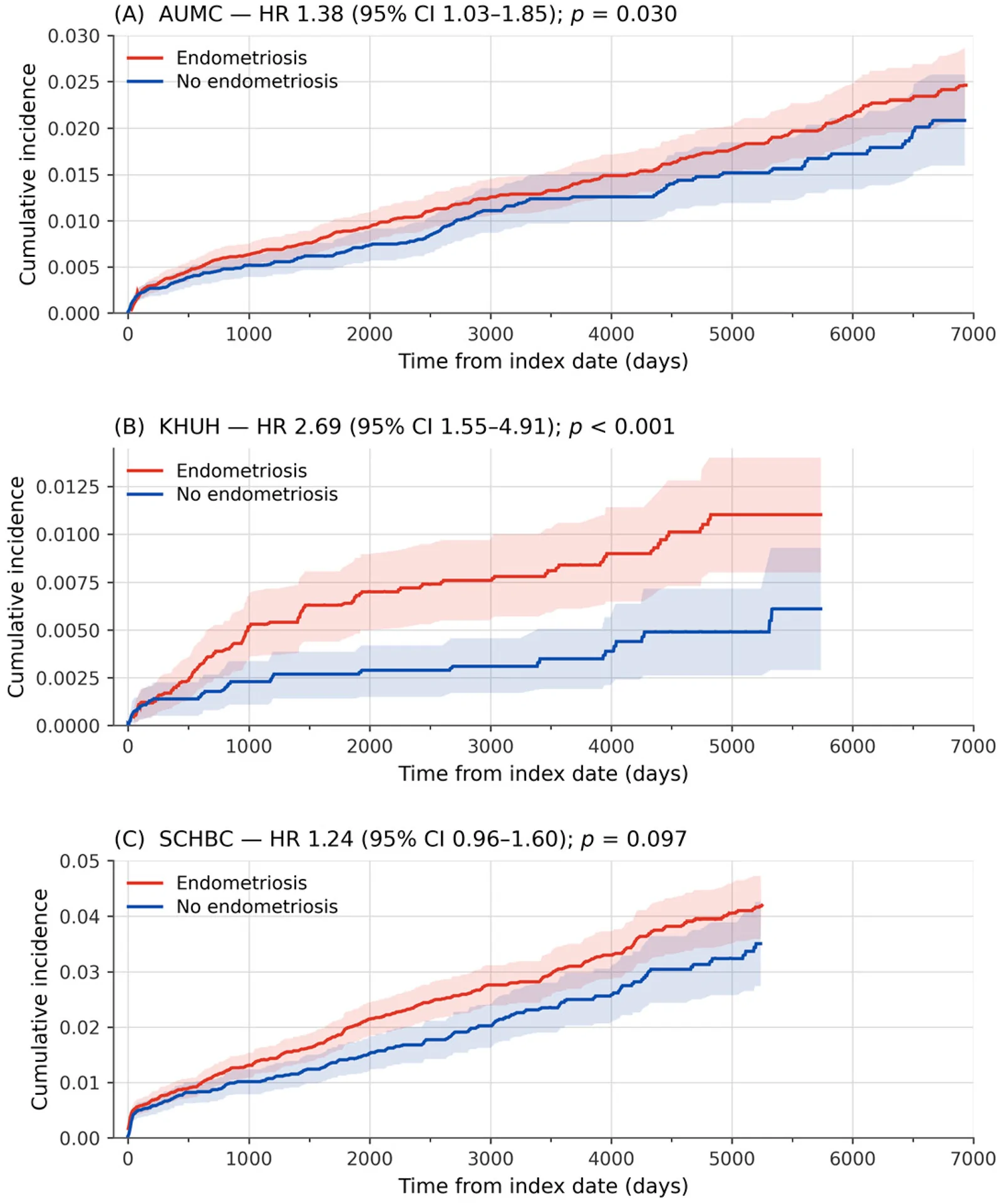
Kaplan–Meier cumulative incidence curves for autoimmune thyroid disease at three representative sites. Sites were selected to represent distinct association patterns: (**A**) AUMC—statistically significant increased risk (HR 1.38, 95% CI 1.03–1.85, *p* = 0.030); (**B**) KHUH—markedly elevated risk (HR 2.69, 95% CI 1.55–4.91, *p* < 0.001); (**C**) SCHBC—trend-level association (HR 1.24, 95% CI 0.96–1.60, *p* = 0.097). Curves show cumulative incidence, computed as 1 − the Kaplan–Meier survival estimate, of newly recorded autoimmune thyroid disease; shaded areas represent 95% confidence intervals. Red: endometriosis cohort; blue: matched no-endometriosis cohort. Time is shown in days from the index date on a common 0–7000-day axis across panels. Sites are shown for illustration; all three panels depict positive or trend-level associations.

Kaplan–Meier curves from three representative sites (AUMC, KHUH, and SCHBC) demonstrated progressive divergence in cumulative incidence between the endometriosis and control groups over the follow-up period (Figure 4). Three sites (CNUH, DCMC, and KWNUH) reported HRs below 1.0 (CNUH 0.53, 95% CI 0.24–1.11; DCMC 0.68, 95% CI 0.37–1.21; KWNUH 0.33, 95% CI 0.02–2.60), and at CNUH, DCMC, HUDU, and KWNUH, the crude incidence rate was lower in the endometriosis cohort than in controls. Two further sites (GNUH, MJH) returned point estimates of exactly 1.0. Site-level results were therefore mixed, with 5 of 12 sites at or below unity, and all estimates—elevated and reduced alike—were imprecise and susceptible to site-level differences in outcome ascertainment.

### 3.4. Sensitivity and Robustness Analyses

In a leave-one-out random-effects meta-analysis (Table S3), the pooled hazard ratio remained above 1.0 in all 12 iterations, ranging from 1.17 to 1.31; the direction of association was therefore robust to exclusion of any single institution, including the two sites reaching individual significance. Omission of KHUH reduced I^2^ from 38.2% to 6.6%, whereas omission of any other site left I^2^ between 21.7% and 44.0%, identifying KHUH empirically as the source of between-site heterogeneity. Omission of either CNUH (HR 0.53) or DCMC (HR 0.68) yielded a pooled estimate that crossed conventional significance (HR 1.30, 95% CI 1.07–1.59, *p* = 0.010 and HR 1.31, 95% CI 1.06–1.62, *p* = 0.014, respectively); this is reported for transparency and indicates that the pooled result is finely balanced and rests on a small number of influential sites rather than indicating that a true positive association exists.

To assess surveillance bias arising from differential follow-up, we reasoned that if the association was generated primarily by the additional detection opportunity afforded by longer observation, sites with a larger follow-up imbalance should show systematically higher hazard ratios. The ratio of mean follow-up (endometriosis: comparator) ranged from 1.27 to 2.01 across sites (mean 1.64). In random-effects meta-regression, the slope of site-specific log hazard ratio on this ratio was 0.085 (SE 0.597; *p* = 0.887), with no monotonic trend (Spearman ρ = 0.31; *p* = 0.32). No relationship between the degree of follow-up imbalance and the magnitude of the site-specific association was therefore detectable.

The E-value for the pooled hazard ratio of 1.22 was 1.74: an unmeasured confounder would need to be associated with both endometriosis and incident autoimmune thyroid disease by a risk ratio of at least 1.74 each, conditional on the measured covariates, to account fully for the observed association. The E-value for the confidence interval limit closest to the null was 1.00 since the interval includes unity.

## 4. Discussion

In this multi-site federated analysis of OMOP-CDM data from 12 Korean tertiary hospitals, we observed a directionally positive but statistically inconclusive association between endometriosis and newly recorded autoimmune thyroid disease. The pooled estimate suggested a modest increase in risk among women with endometriosis but did not reach conventional statistical significance (pooled HR 1.22, 95% CI 0.97–1.53; *p* = 0.090). Nevertheless, the direction of association was preserved in the majority of participating sites, with 7 of 12 sites reporting HRs above 1.0.

Our findings are directionally consistent with prior epidemiological evidence. In Korean data, Yuk et al. [8] reported a significant association between endometriosis and Graves’ disease (OR 2.52; 95% CI 1.30–4.88). Two recent large-scale studies further support this direction: Aziz et al. [20] demonstrated significantly elevated odds of Hashimoto’s thyroiditis (OR 2.25–2.77) and Graves’ disease (OR 1.46–4.55) in women with endometriosis using US administrative claims data, and a TriNetX-based 20-year cohort study [21] reported significantly elevated risks of thyroiditis (HR 1.32; 95% CI 1.17–1.50) and Graves’ disease (HR 1.27; 95% CI 1.04–1.56) independent of treatment modality. Although our pooled HR of 1.22 was not statistically significant, its magnitude falls within the lower range of these prior estimates. The statistically inconclusive result is also consistent with prior systematic reviews reporting heterogeneity across studies of endometriosis and autoimmune thyroid disease, likely reflecting differences in study design, population structure, outcome definitions, and ascertainment methods [8,22]. Therefore, our findings should be interpreted as multi-site real-world evidence that is concordant with, but not confirmatory of, the previously reported endometriosis–thyroid autoimmunity association.

Two further studies warrant specific consideration. Şerifoğlu et al. [23], in a prospective series of 102 women undergoing surgery for benign gynecological disease (51 with and 51 without endometriosis), reported an association between endometrioma diameter and anti-thyroid peroxidase antibody titer—evidence from a surgically confirmed cohort with direct antibody measurement and therefore of a kind the present study cannot supply. Kirkegaard et al. [22] caution that associations between thyroid and gynecological conditions are particularly vulnerable to detection bias arising from differential healthcare contact, a caution that aligns directly with the principal limitation of our own design.

The case against a shared mechanism should also be stated. Endometriosis is an estrogen-dependent inflammatory condition whose dominant immunological features are impaired natural killer cell cytotoxicity and macrophage-mediated lesion maintenance, whereas Hashimoto’s thyroiditis and Graves’ disease are organ-specific autoimmune conditions driven by antigen-specific T- and B-cell responses to thyroid peroxidase, thyroglobulin, and the TSH receptor, with strong HLA and CTLA-4 associations that endometriosis does not share. Shared female preponderance and a shared reproductive-age incidence peak provide ample opportunity for confounding and co-detection independent of any common pathway. The mechanistic evidence discussed below therefore establishes plausibility rather than a demonstrated mechanism.

Several biological mechanisms may plausibly link endometriosis and autoimmune thyroid disease. Endometriosis is increasingly understood as a systemic inflammatory condition rather than a disease confined to the pelvis and has been associated with multiple autoimmune comorbidities [24,25,26]. Both endometriosis and autoimmune thyroid disease involve immune dysregulation characterized by Th1-skewed cytokine profiles, altered regulatory T-cell function, and enhanced autoantibody production [8,9]. Peyneau et al. [27] demonstrated that endometriotic lesions express functional TSH receptors and that thyroid hormones may directly modulate ectopic endometrial cell proliferation, suggesting potential hormonal–immunological crosstalk between the thyroid axis and endometriotic tissue. In addition, thyroid autoantibody positivity, including anti-TPO antibodies and elevated TSH receptor antibody titers, has been reported in women with endometriosis or in reproductive contexts involving thyroid autoimmunity [28,29,30]. These findings provide a mechanistic basis for the observed direction of association, although they do not establish causality. These observations are hypothesis-generating; none constitute evidence of a causal pathway operating at a population scale.

The statistically inconclusive pooled estimate in the present study should be interpreted in light of several CDM-specific and clinical data limitations. First, the mean observation time was substantially longer in the endometriosis cohort than in controls (2693 vs. 1677 days), likely reflecting higher healthcare utilization among women with endometriosis. Although Cox regression accounts for differential follow-up time, differences in healthcare contact may still influence the likelihood of thyroid disease detection and coding. Second, autoimmune thyroid disease may be inconsistently captured across institutions because Hashimoto’s thyroiditis and Graves’ disease can be coded differently depending on local diagnostic practices, thyroid function testing patterns, and the availability or use of antibody testing. Third, the number of outcome events was small in several sites, which may have contributed to unstable site-specific estimates. Thus, the inconclusive pooled result may reflect a combination of modest effect size, outcome misclassification, site-level coding heterogeneity, differential healthcare utilization, and limited event numbers, rather than providing definitive evidence either for or against the association. Two further considerations bear on interpretation. The pooled incidence rate ratio was 1.14 (1.51 vs. 1.32 per 1000 patient-years)—a modest difference, smaller than the pooled hazard ratio of 1.22, and substantially smaller than the 1.61-fold difference in mean follow-up duration; because incidence rates are expressed per unit of person-time, longer follow-up in the endometriosis cohort does not by itself inflate them. The pattern was, moreover, not uniform, since in four sites the crude incidence rate was lower in the endometriosis cohort, which a uniform biological effect would not produce. The 95% confidence interval (0.97–1.53) is compatible both with no association and with a clinically meaningful 50% increase in the rate of newly recorded autoimmune thyroid disease. A non-significant result at this sample size should therefore not be read as evidence of absence; equally, the interval’s inclusion of unity means that the present data do not establish the association.

Between-site heterogeneity was moderate (I^2^ = 38.2%), which is expected in a federated multi-institutional EHR-based analysis. Differences in institutional patient populations, referral patterns, thyroid disease diagnostic practices, and CDM mapping quality may all contribute to this heterogeneity. The leave-one-out analysis (Section 3.4; Table S3) identifies KHUH empirically as the source of this heterogeneity: its omission reduced I^2^ to 6.6%, whereas the omission of any other site left I^2^ between 21.7% and 44.0%. As to why, the data support a parsimonious explanation. KHUH had the lowest comparator-cohort incidence rate of any site (0.45 per 1000 patient-years, against a pooled comparator rate of 1.32 and a site range of 0.33–2.95), while its endometriosis-cohort rate (0.87) was also below the pooled figure. The elevated hazard ratio at this site therefore arises from an unusually low comparator rate rather than from an unusually high rate among women with endometriosis, which is more consistent with site-specific ascertainment or coding completeness in the comparator cohort than with a distinct biological effect; we cannot verify this without site-level chart review. The sites with hazard ratios below 1.0 warrant the same caution and the same standard of evidence: CNUH and DCMC recorded lower crude incidence in the endometriosis cohort, and their estimates are no less susceptible to institutional ascertainment differences than the elevated ones. With 5 of 12 sites at or below unity, the site-level evidence is best described as mixed. A further caveat applies to the pooling itself: a random-effects model combining twelve sites of markedly unequal size yields a τ^2^ estimate that is itself imprecise.

The differential follow-up between cohorts requires quantitative rather than qualitative treatment, and the analyses reported in Section 3.4 provide it. Across the 12 sites, the ratio of mean follow-up varied appreciably (1.27–2.01) yet showed no association with the site-specific log hazard ratio (meta-regression slope 0.085, SE 0.597, *p* = 0.887). Had differential observation time been the principal driver of the association, a positive gradient would be expected; none was detectable. Together with the person-time basis of the incidence rates and the E-value of 1.74 for unmeasured confounding, this bounds—but does not eliminate—the two leading non-causal explanations. Specifically, these analyses address detection opportunity arising from follow-up duration, not differential intensity of thyroid testing per unit of follow-up time. Women already engaged in tertiary gynecological care may undergo more frequent thyroid function testing and present more coding opportunities per unit time, and neither the number of thyroid function tests nor outpatient visit frequency was captured in the propensity score. Equivalent ascertainment between cohorts has therefore not been demonstrated, and this remains the principal threat to the validity of the present estimate. We accordingly draw no clinical inference from these findings.

This study has several strengths. First, the federated OMOP-CDM design enabled a multi-site analysis across 143,238 matched participants without sharing patient-level data, supporting privacy-preserving real-world evidence generation. Second, the use of a common analytic protocol and standardized CDM structure allowed comparable cohort construction and outcome assessment across 12 tertiary hospitals. Third, site-specific effect estimates and random-effects meta-analysis enabled evaluation of between-site heterogeneity, which is difficult to assess in single-database claims studies. In this sense, the novelty of the present study lies not only in evaluating the endometriosis–autoimmune thyroid disease association in a Korean population, but also in testing the reproducibility and robustness of this association across multiple real-world clinical data sources. The use of hypertension and selected Charlson comorbidity index components in propensity score matching also provided consistent baseline comorbidity adjustment across institutions.

Several limitations should be acknowledged. First, endometriosis was defined using structured diagnostic codes rather than uniform surgical or histopathologic confirmation; therefore, some degree of exposure misclassification is possible, and disease severity or lesion subtype could not be assessed. In addition, because a sufficient washout period for endometriosis diagnosis was not applied, some patients may have represented prevalent rather than newly diagnosed cases. Second, autoimmune thyroid disease was defined using diagnostic codes within the OMOP-CDM framework; therefore, outcome misclassification is possible, particularly for subclinical Hashimoto’s thyroiditis or antibody-positive thyroid autoimmunity that may not be consistently coded across institutions. The lack of thyroid autoantibody measurements, including anti-TPO, anti-thyroglobulin, and TSH receptor antibodies, as well as thyroid function tests such as TSH and free T4, further limited assessment of subclinical disease and disease severity. Third, residual detection bias and unmeasured confounding cannot be excluded because thyroid testing frequency, outpatient visit frequency, infertility status, and gynecologic healthcare utilization were not fully captured in the propensity score model. Finally, the analysis was restricted to tertiary academic hospitals, which may limit generalizability to community-based populations. Several further limitations follow from the reviewers’ points and are stated here explicitly. The composite outcome combines Hashimoto’s thyroiditis and Graves’ disease, which differ in autoantigen target, in thyroid functional consequence, and in their relationship to smoking—positive for Graves’ disease and inverse for Hashimoto’s thyroiditis—so that a composite may mask divergent or even opposing subtype-specific associations; disaggregation was not possible here because the federated design transfers only aggregate site-level statistics, and re-execution at all 12 institutions would be required. The 365-day pre-index washout is a compromise between prevalent-case exclusion and cohort retention, constrained by the site-specific data availability periods (Table S1); women with autoimmune thyroid disease diagnosed earlier but without a code recorded within the washout window would be misclassified as incident cases, and slowly evolving subclinical disease beginning before the index date would be captured as an incident event. The index events also differ in clinical meaning between cohorts: a first endometriosis diagnosis marks a point of intensified gynecological engagement, whereas a randomly selected outpatient visit does not. The random-visit anchor is the standard comparator assignment in the OHDSI Cohort Method framework, chosen because cohort-entry anchoring would introduce immortal time bias and fixed-date anchoring would remove the calendar-time distribution that the propensity score must balance; index calendar year was included as a covariate for this reason. Nonetheless, this asymmetry is, in our judgment, the same phenomenon that produces the differential follow-up described above and the most likely non-causal explanation for a modest positive association. Finally, socioeconomic status is not represented in hospital-derived CDM instances, and infertility—both a consequence of endometriosis and a common route to its diagnosis—requires causal rather than routine adjustment, since conditioning on it risks collider bias.

## 5. Conclusions

This multi-site OMOP-CDM analysis observed a directionally positive but statistically inconclusive association between endometriosis and newly recorded autoimmune thyroid disease. The pooled estimate was robust in the direction of the omission of any single site, and neither differential follow-up duration nor plausible unmeasured confounding of ordinary magnitude offered a sufficient alternative explanation; equally, the confidence interval includes unity, and site-level results were mixed. These findings are hypothesis-generating and do not support any change in the clinical evaluation of women with endometriosis. Resolving the question will require prospective cohorts with protocol-specified thyroid assessment—anti-thyroid peroxidase, anti-thyroglobulin, and TSH receptor antibodies together with TSH and free T4—measured at fixed intervals independent of symptom-driven testing, harmonized follow-up between exposed and unexposed groups, outcome adjudication that does not depend on administrative coding, and analysis of Hashimoto’s thyroiditis and Graves’ disease as distinct endpoints.

## Figures and Tables

**Table 1:** Baseline characteristics of women with and without endometriosis before and after 1:1 propensity score matching across 12 OMOP-CDM sites.

	Before PS Matching			After PS Matching		
Variable	w/o ES (*n* = 2,863,891)	with ES (*n* = 74,433)	SMD	w/o ES (*n* = 71,619)	with ES (*n* = 71,619)	SMD
Age group, *n* (%)						
18–24	515,034 (17.98)	4988 (6.70)	−0.348	5185 (7.24)	5067 (7.07)	−0.006
25–29	386,005 (13.48)	7951 (10.68)	−0.086	7858 (10.97)	7864 (10.98)	<0.001
30–34	397,692 (13.89)	11,390 (15.30)	0.040	11,387 (15.90)	11,155 (15.58)	−0.009
35–39	363,326 (12.69)	14,287 (19.19)	0.178	14,145 (19.75)	13,752 (19.20)	−0.013
40–44	325,270 (11.36)	15,222 (20.45)	0.251	16,176 (22.59)	15,975 (22.31)	−0.006
45–49	316,667 (11.06)	13,236 (17.78)	0.192	11,921 (16.65)	12,401 (17.32)	0.018
50–54	295,073 (10.30)	5030 (6.76)	−0.127	4358 (6.08)	4723 (6.59)	0.021
55–60	217,630 (7.60)	723 (0.97)	−0.332	589 (0.82)	682 (0.95)	0.018
Comorbidities (Charlson), *n* (%)						
Hypertension	13,044 (0.46)	402 (0.54)	0.012	280 (0.39)	392 (0.55)	0.023
Cerebrovascular disease	24,182 (0.84)	524 (0.70)	−0.016	402 (0.56)	505 (0.71)	0.018
Chronic pulmonary disease	7372 (0.26)	129 (0.17)	−0.018	73 (0.10)	123 (0.17)	0.019
Peptic ulcer disease	2544 (0.09)	4 (0.01)	−0.038	45 (0.06)	3 (0.00)	−0.032
Diabetes	12,057 (0.42)	245 (0.33)	−0.015	190 (0.27)	249 (0.35)	0.015
Diabetes with chronic complications	207 (0.01)	7 (0.01)	0.002	13 (0.02)	13 (0.02)	<0.001
Renal disease	2172 (0.08)	41 (0.06)	−0.008	24 (0.03)	40 (0.06)	0.010
Any malignancy	25,057 (0.87)	681 (0.91)	0.004	790 (1.10)	728 (1.02)	−0.008
Mild liver disease	4864 (0.17)	71 (0.10)	−0.020	93 (0.13)	73 (0.10)	−0.008
Moderate to severe liver disease	233 (0.01)	11 (0.02)	0.007	11 (0.01)	11 (0.01)	<0.001

Abbreviations: ES = endometriosis; PS = propensity score; SMD = standardized mean difference. All Charlson comorbidity index components were used as covariates in propensity score estimation. All post-matching SMDs were <0.10. For continuous variables, SMD = ($\overset{-}{x}$1 − $\overset{-}{x}$2)/√[(s1^2^ + s2^2^)/2]. For binary variables, SMD = (p1 − p2)/√{[p1(1 − p1) + p2(1 − p2)]/2}, where p1 and p2 denote the proportions in the endometriosis and comparator cohorts, respectively.

**Table 2:** Incidence rates and hazard ratios for autoimmune thyroid disease in women with vs. without endometriosis (1:1 PS-matched cohorts, random-effects meta-analysis).

Hospital	Number of Subjects		Mean Follow-Up Time (Days)		Number of Outcome Events		Incidence Rate per 1000 Patient-Years		HR (95% CI)	*p* Value
	w/o ES	with ES	w/o ES	with ES	w/o ES	with ES	w/o ES	with ES		
AUMC	17,945	17,945	1850.2	3199.5	118	225	1.30	1.43	1.38 (1.03–1.85)	0.030
CNUH	5817	5817	1457.1	2017.3	23	20	0.99	0.62	0.53 (0.24–1.11)	0.105
DCMC	6663	6663	1553.7	2334.0	42	42	1.48	0.99	0.68 (0.37–1.21)	0.195
GNUH	1385	1385	874.9	1342.0	7	13	2.11	2.55	1.00 (0.34–2.92)	1.000
HUDU	3626	3626	1203.2	1999.5	7	10	0.59	0.50	1.33 (0.46–4.05)	0.603
ISH	2809	2809	1157.5	2058.5	10	21	1.12	1.33	1.33 (0.56–3.27)	0.521
KHUH	7727	7727	2622.1	3331.6	25	61	0.45	0.87	2.69 (1.55–4.91)	<0.001
KWNUH	2335	2335	2391.4	4102.8	5	8	0.33	0.31	0.33 (0.02–2.60)	0.395
MJH	1664	1664	1824.3	2979.0	6	9	0.72	0.66	1.00 (0.28–3.60)	1.000
PNUH	4837	4837	1171.6	2089.0	19	31	1.22	1.12	1.31 (0.64–2.75)	0.471
SCHBC	10,635	10,635	1677.1	2735.1	144	273	2.95	3.43	1.24 (0.96–1.60)	0.097
SCHSU	6176	6176	1115.3	2240.0	29	87	1.54	2.30	1.45 (0.85–2.53)	0.179
Pooled (random-effects, k = 12)	71,619	71,619	1677.3	2693.4	435	800	1.32	1.51	1.22 (0.97–1.53)	0.090

Abbreviations: ES = endometriosis; HR = hazard ratio. Number of subjects: cohort size after 1:1 PS matching. Mean follow-up = total person-days/number of subjects. Site abbreviations are defined in Table S1. Pooled HR: DerSimonian–Laird random-effects meta-analysis of site-specific Cox proportional hazards ratios. Time-at-risk: index date +1 day to the earliest of outcome event, end of observation, or death.

## Data Availability

The patient-level data underlying this study are not publicly available because of privacy and institutional restrictions. The study used de-identified OMOP-CDM data held locally by the participating institutions; only aggregate summary statistics were shared for meta-analytic pooling. The analytic definitions may be made available by the corresponding author upon reasonable request, subject to institutional approval.

## Supplementary Materials

The following supporting information can be downloaded at: https://www.mdpi.com/article/10.3390/jcm15176919/s1, Table S1, site abbreviations and OMOP-CDM data availability periods; Table S2, OMOP-CDM concept set definitions; Table S3, leave-one-out random-effects meta-analysis across the 12 participating sites.

## Abbreviations

The following abbreviations are used in this manuscript:

anti-TPO	anti-thyroid peroxidase
CDM	Common Data Model
CI	confidence interval
EHR	electronic health record
ES	endometriosis
HR	hazard ratio
IR	incidence rate
KCD	Korean Classification of Diseases
KCD7	Korean Classification of Diseases, seventh revision
OHDSI	Observational Health Data Sciences and Informatics
OMOP	Observational Medical Outcomes Partnership
OR	odds ratio
PS	propensity score
SMD	standardized mean difference
TRAb	thyrotropin receptor antibody
TSH	thyroid-stimulating hormone
