# Supplementary material for: Association Between Endometriosis and Autoimmune Thyroid Disease Using a Multicenter Observational Medical Outcomes Partnership (OMOP) Common Data Model
Source: J Clin Med. 2026 Sep 7;15(17):6919. doi: 10.3390/jcm15176919 (PMC13566532; doi:10.3390/jcm15176919)
Supplement: Supplementary file 1 [file jcm-15-06919-s001.zip › jcm-4484523-supplementary.pdf]

Table S1. Site abbreviations and OMOP-CDM data availability periods.

Site abbreviation	Participating hospital	OMOP-CDM data availability period
AUMC	Ajou University Medical Center	1994-07 – 2026-04
CNUH	Chungnam National University Hospital	2013-05 – 2026-04
DCMC	Daegu Catholic University Medical Center	2005-01 – 2026-04
GNUH	Gyeongsang National University Changwon Hospital	2016-03 – 2026-04
HUDU	Hallym University Dongtan Sacred Heart Hospital	2009-12 – 2023-05
ISH	INTL St. Mary's Hospital	2014-03 – 2026-04
KHUH	Kyung Hee University Hospital at Gangdong	2006-06 – 2026-04
KWNUH	Kangwon National University Hospital	2003-01 – 2026-04
MJH	Myongji Hospital	2003-10 – 2026-04
PNUH	Pusan National University Hospital	2011-01 – 2026-05
SCHBC	Soonchunhyang University Bucheon Hospital	2003-05 – 2024-04
SCHSU	Soonchunhyang University Seoul Hospital	2001-01 – 2024-04

Table S2. OMOP-CDM concept set definitions.

Concept Set	Role in Study	Concept ID	Concept Name	Vocab
Endometriosis (Exposure)	Target cohort definition; Comparator cohort exclusion	433527	Endometriosis (clinical)	SNOMED
		42494932	Endometriosis of uterus	KCD7
		42494933	Endometriosis of ovary	KCD7
		42494934	Endometriosis of fallopian tube	KCD7
		42494935	Endometriosis of pelvic peritoneum	KCD7
		42494936	Endometriosis of rectovaginal septum and vagina	KCD7
		42494937	Endometriosis of intestine	KCD7
		42494938	Endometriosis in cutaneous scar	KCD7
		42494939	Other endometriosis	KCD7
		42494940	Endometriosis, unspecified	KCD7
Autoimmune thyroid disease (Outcome)	1) Primary outcome (newly recorded post-index event for outcome analysis) 2) Baseline exclusion (prior autoimmune thyroid disease within 365 days before index date -> excluded from both cohorts)	42485936	Autoimmune thyroiditis (including Hashimoto's thyroiditis)	KCD7
		42485924	Thyrotoxicosis with diffuse goiter (Graves' disease)	KCD7

All codes belong to the Condition domain. Abbreviations: CDM = Common Data Model; KCD7 = Korean Classification of Diseases version 7; OMOP = Observational Medical Outcomes Partnership; SNOMED = SNOMED Clinical Terms.All concept sets were applied with 'Include Descendants = YES', capturing all hierarchical child codes within the OMOP vocabulary hierarchy.

Table S3. Leave-one-out random-effects meta-analysis of the association between endometriosis and newly recorded autoimmune thyroid disease across 12 OMOP-CDM sites.

Site omitted	Pooled HR	95% CI	P value	I^2 (%)
None (all 12 sites)	1.22	0.97–1.53	0.090	38.2
AUMC	1.17	0.89–1.56	0.266	42.4
CNUH	1.30	1.07–1.59	0.010	21.7
DCMC	1.31	1.06–1.62	0.014	25.5
GNUH	1.23	0.96–1.56	0.103	43.5
HUDU	1.21	0.95–1.55	0.128	44.0
ISH	1.21	0.94–1.55	0.137	44.0

**KHUH**	**1.17**	**0.99–1.40**	**0.068**	**6.6**
KWNUH	1.23	0.98–1.55	0.075	40.1
MJH	1.22	0.96–1.56	0.104	43.6
PNUH	1.21	0.94–1.55	0.146	44.0
SCHBC	1.19	0.89–1.60	0.233	44.0
SCHSU	1.19	0.92–1.54	0.193	43.1

Each row shows the DerSimonian–Laird random-effects pooled estimate recomputed after omitting the indicated site. Site-specific estimates are those reported in Table 2 of the main text; no re-execution of the distributed protocol was required. The pooled hazard ratio remained above 1.0 in all 12 iterations (range 1.17–1.31). Omission of KHUH reduced I^2 from 38.2% to 6.6%, whereas omission of any other site left I^2 between 21.7% and 44.0%. Omission of CNUH or DCMC — the two sites with the lowest hazard ratios — yielded a pooled estimate crossing conventional significance, indicating that the pooled result is finely balanced and rests on a small number of influential sites. Abbreviations: CI = confidence interval; HR = hazard ratio; OMOP-CDM = Observational Medical Outcomes Partnership Common Data Model. The all-sites row reports the primary analysis (Section 3.3). Leave-one-out rows were recomputed by DerSimonian–Laird pooling of the site-specific hazard ratios and confidence intervals as reported in Table 2 (rounded to two decimal places); recomputing the all-sites estimate from these rounded values reproduces the primary result to within rounding (HR 1.22, 95% CI 0.97–1.53; $p = 0.093$; $I^2 = 38.4\%$).
